# Clinical Approach to Neuroendocrine Neoplasm Associated With Ovarian Teratoma

**DOI:** 10.3389/fendo.2021.770266

**Published:** 2021-11-30

**Authors:** Marta Opalińska, Anna Sowa-Staszczak, Helena Olearska, Magdalena Ulatowska-Bialas, Aleksandra Gilis-Januszewska, Alicja Hubalewska-Dydejczyk

**Affiliations:** ^1^ Nuclear Medicine Unit, Department of Endocrinology Department of Endocrinology, Oncological Endocrinology and Nuclear Medicine, University Hospital, Kraków, Poland; ^2^ Chair and Department of Endocrinology, Jagiellonian University Medical College, Kraków, Poland; ^3^ Chair of Pathomorphology, Jagiellonian University Medical College, Kraków, Poland

**Keywords:** NET, teratoma, ovary tumor, neuroendocrine tumors, ovarian carcinoid

## Abstract

**Background:**

Neuroendocrine neoplasms are a heterogeneous group of cancers that develop from enterochromaffin cells of the diffuse endocrine system, with an increase in incidents over the last years. Ovarian neuroendocrine tumors (NET) are rare neoplasms, comprising 0.1% of all ovarian neoplasms and less than 5% of all neuroendocrine tumors. They may arise alone (as monodermal, specialized teratoma – ovarian carcinoid) or as a part of other ovarian lesion: cystic mature or immature teratomas. Due to the rarity and limited amount of such cases reported in the literature, there is no consensus on diagnostic and therapeutic procedures in this group of patients.

**Materials and Methods:**

The group of 10 patients at the age of 19 to 77 years (mean 42.8 ± 17.9), diagnosed with unilateral NET within ovarian teratoma were analyzed. The histopathological type of tumor, progression free survival after surgical treatment and presence of hormonally active syndrome were assessed.

**Results:**

70% (n=7) of patients was diagnosed with mature cystic teratomas containing NET component and 30% (n=3) with monodermal teratoma (strumal carcinoid). All cases of monodermal teratomas were found in women at premenopausal age. Determined Ki67 ranged from 2% to 9%. Ninety percent of lesions (n=9) stained positive for synaptophysin and chromogranin, while markers: CK20, CK7, TTF-1 and CDX2 were negative in all cases, which ruled out their metastatic nature. None of the patients presented with carcinoid syndrome. All followed-up patients remain progression-free, which confirms surgical intervention being a crucial and sufficient method of treatment.

**Conclusions:**

The prognosis and clinical behavior of NETs associated with ovarian teratomas are good with long progression-free survival.

## Highlights

- NET associated with ovarian teratomas are rare findings, possible in women of any age
- Surgical treatment is crucial and usually sufficient as a method of radical treatment of NETs associated with ovarian teratomas
- The prognosis of NETs associated with ovarian teratomas seem to be very good, with long progression-free survival, although there are no specific guidelines for follow-up

## Introduction

Ovarian teratomas constitute frequent gynecological finding. They include mature cystic teratomas, immature teratomas and monodermal (specialized) teratomas. Mature cystic teratoma typically contains mature tissues of ecto-, meso- and endodermal origin. Immature (malignant) teratoma contains both mature and immature tissues e.g. tissue which resembles immature embryonal tissue (1).

On the other side neuroendocrine neoplasms (NENs) which are a heterogeneous group of neoplasm originating from enterochromaffin cells scattered throughout the body. They are mostly found in gastrointestinal and respiratory tracts with an estimated increasing incidence rate of 6.9/100,000 (2). Most of them occur sporadically, but there is known association with various genetic disorders, mainly MEN 1 and MEN 4 syndrome (3).

According to WHO classification of digestive system tumors, NENs might be highly or moderately differentiated called neuroendocrine tumors (NETs G1 or G2 with Ki 67 index below 20%) and poorly differentiated with Ki67 over 20% (NET G3 with still organoid histology or NEC (neuroendocrine carcinoma) without organoid histology (4). The degree of differentiation determines the prognosis and the method of treatment.

The new WHO classification of gynecologic NENs (2020) distinguish only well-differentiated tumors (NETs) and poorly differentiated carcinomas (NEC) and for neuroendocrine tumors of ovary WHO still recommends the use of “ovarian carcinoid” term instead of well-differentiated neuroendocrine tumor (1).

In general ovarian NETs are uncommon. They comprise only 0.1% of all ovarian neoplasms and less than 5% of all neuroendocrine tumors (5). Only part of them are primary ovarian tumors, while rest have metastatic origin usually from NETs of gastrointestinal or respiratory systems. Other gynecological organs like cervix, endometrium vagina or vulva are less frequently affected by NEN. In those localizations most NENs is diagnosed as NECs (6–8).

Most of ovarian NETs originate from monodermal teratomas. In general monodermal subtypes of teratomas are built only from one tissue type. In case of being built of a thyroid tissue they are called struma ovarii. If built from neuroendocrine tissue they are called ovarian carcinoid (insular, trabecular or mucinous type) (9). A presence of struma ovarii and carcinoid tissue in the same ovary is called a strumal carcinoid. The most common forms of ovarian carcinoid are insular and strumal type while trabecular and mucinous carcinoid are very rare (4). Most ovarian carcinoids of insular and trabecular type express chromogranin, synaptophysin and CD56, although trabecular one may be chromogranin negative. Insular and mucinous carcinoids may be positive with CDX2 (10). Both insular and trabecular carcinoids are typically CK7 positive and CK20 negative. Mucinous carcinoids are often CK20 positive and CK7 negative. Strumal carcinoids exhibit positive staining with neuroendocrine markers (carcinoid component) and thyroglobulin and thyroid transcription factor (TTF1) (thyroid component). The Ki67 proliferation index in primary ovarian carcinoid tumors of insular, trabecular and strumal types is usually less than 1% (5). The expression of somatostatin receptors on NEN cell surface (SSTR 2-5) (seen usually on well-differentiated NETs) enables diagnostic (imaging with 99mTc/68GaHYNIC/DOTA-peptides in SPECT/CT or PET/CT techniques respectively) and therapeutic procedures with the use of somatostatin analogues (peptide receptor radiation therapy (PRRT) with 177Lu or 90Y/177Lu-DOTA-TATE/TOC) (11).

## Material and Methods

The aim of the study was to assess the clinical outcome of patients with postoperative diagnosis of neuroendocrine tumors (NET) in ovarian teratoma.

The study was designed as a retrospective analysis. Eligible patients were those who met main inclusion criteria: diagnose with NET in ovary teratoma which was histopathology confirmed according to WHO classification (2017 or 2019).

All patients meet the inclusion criteria were identified among the group of about 600 patients referred to our center during 2013-2020 (since the time of introduction of electronic documentation) due to suspicion of NET or confirmed NET diagnose. The identification was done by screening consecutive patients’ records and collection of clinical/outcome data. Histopathological results were confirmed locally by pathologists with an expertise in the field of NETs blinded from clinical data.

Study variables included initial patients data, operation characteristics, postoperative complications, the course of disease and follow-up. Those data were extracted from the patient electronic records. The follow-up time was defined as the time from the patient’s first visit to last visit.

Histological differentiation was assessed according to WHO classification for NET (2017, 2019), based on the morphological mitotic index (G1 <2 in 10 large visual fields (HPF), G2: 2-20/10HPF, and G3: > 20/10HPF) or immunohistochemically assessed tumor proliferative activity according to the Ki-67 index (G1 <3%, G2 3-20%, G3 > 20%). In cases where the mitotic index differed from the Ki-67 index, a higher index was used.

The surgical material was fixed in formalin, routinely processed and paraffin embedded. All cases were revaluated by a two pathologist experienced in a ovarian pathology. In each case, a single section including well-preserved part of tumor containing suspected neuroendocrine tissue was chosen. From this tissue block, 3 μm sections were prepared and immunohistochemistry with chromogranin [anti-chromogranin A antibody, Ventana (LK2H10)], synaptophysin [anti-synaptophysin antibody, Ventana (MRQ-40)] and MIB antibody was performed by a standard method. Briefly, the slides were dewaxed, rehydrated and incubated in 3% peroxide solution for 10 minutes to block endogenous peroxidase activity. Antigen retrieval was carried out by microwaving in citrate buffer (pH 6.0) for 5 minutes at 700 W, then for 5 minutes at 600 The LabVision (Thermo Fisher Scientific, USA) detection system was used. 3-amino-9-ethylcarbasole served as the chromogen. The slides were counterstained with Mayer’s hematoxylin (DAKO, Denmark).

The follow-up examination included gynecological examinations with transvaginal ultrasound every 6-12 moths. Transabdominal ultrasound or abdominal and pelvic CT were performed 4-6 months after surgery, later every 12 month. Chromogranin A level was assessed on the first postoperative visit and in 6-12 months periods.

### Statistical Analysis

Demographic and clinical characteristics were analyzed by producing tables of frequency for categorical variables and by calculation of the median and range for continuous variables. Length of follow-up is presented for the group as range and median value in months, from the time of curative surgery until the last follow-up appointment. IBM SPSS Statistics 26 was employed for the statistical analysis.

## Results

There were 10 patient eligible to include to the analysis. The mean age of patients at diagnosis was 42.8 ± 17.9 years (range 19–77). Seven of them were of reproductive age, and 3 were at menopause. In premenopausal patients, lesions were revealed in the majority of cases in a routine gynecological examination, in one case during pregnancy (ended later by caesarean section). In postmenopausal patients, the ovarian tumor was discovered while diagnosing specific complains either related to the disease itself (abdominal pain) or not (syncope). Two patients had a positive history of breast cancer treated surgically with subsequent radiotherapy, in oncological remission. None of the patients presented with carcinoid syndrome.

Each patient had a unilateral lesion and 70% of them (n=7) were located in the left ovary. The side did not correlate with an age of patients. All patients were operated not later than 6 months from diagnosis with laparoscopy or laparotomy. There were no complications in the course of surgeries or postoperative period. Postoperative imaging studies (CT scans and transvaginal ultrasounds) showed no abnormalities. In pathology the most common diagnosis was neuroendocrine tumor within ovarian teratoma present in 70% (n=7) of patients. Another 30% (n=3) were diagnosed with monodermal teratoma ovarian carcinoid. Tumors were usually large in their largest dimension. The mean teratoma diameter was 94.4mm [range 30-195)], however, neuroendocrine components in them were significantly smaller, 5-15 mm. No correlation was found between the size of tumor and a size of NET lesion or the affected side.

In premenopausal women there were 4 cases of NET within mature ovarian teratoma and 3 cases of monodermal teratoma with strumal carcinoid. In all cases of postmenopausal women (n=3) the NET within mature ovarian teratoma were diagnosed.

**Figure 1:**
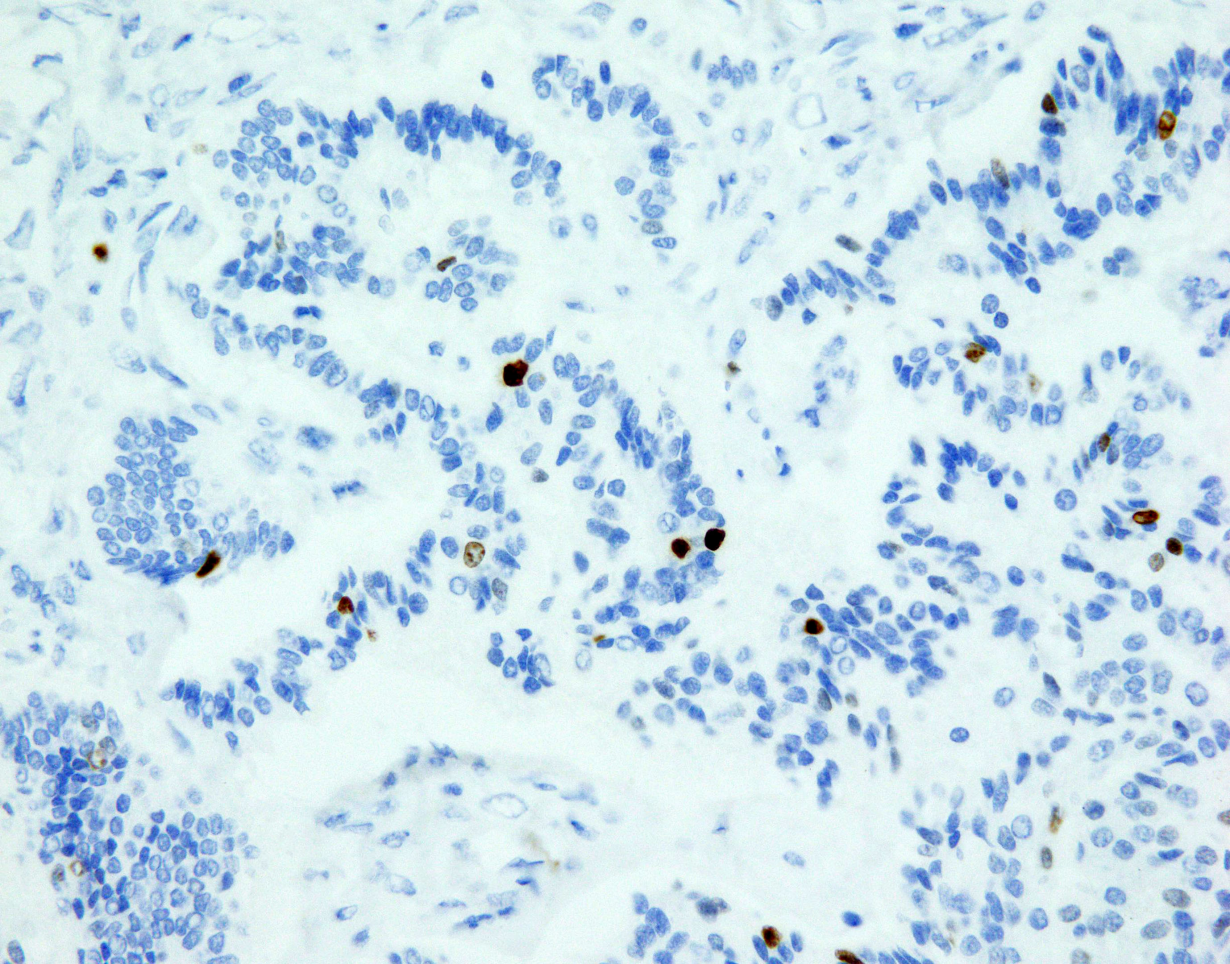
NET focus in teratoma. Ki67 expression in NET foci of teratoma, zoom 40x. Immunohistochemical staining Ki67: proliferative activity around 1%.

**Figure 2:**
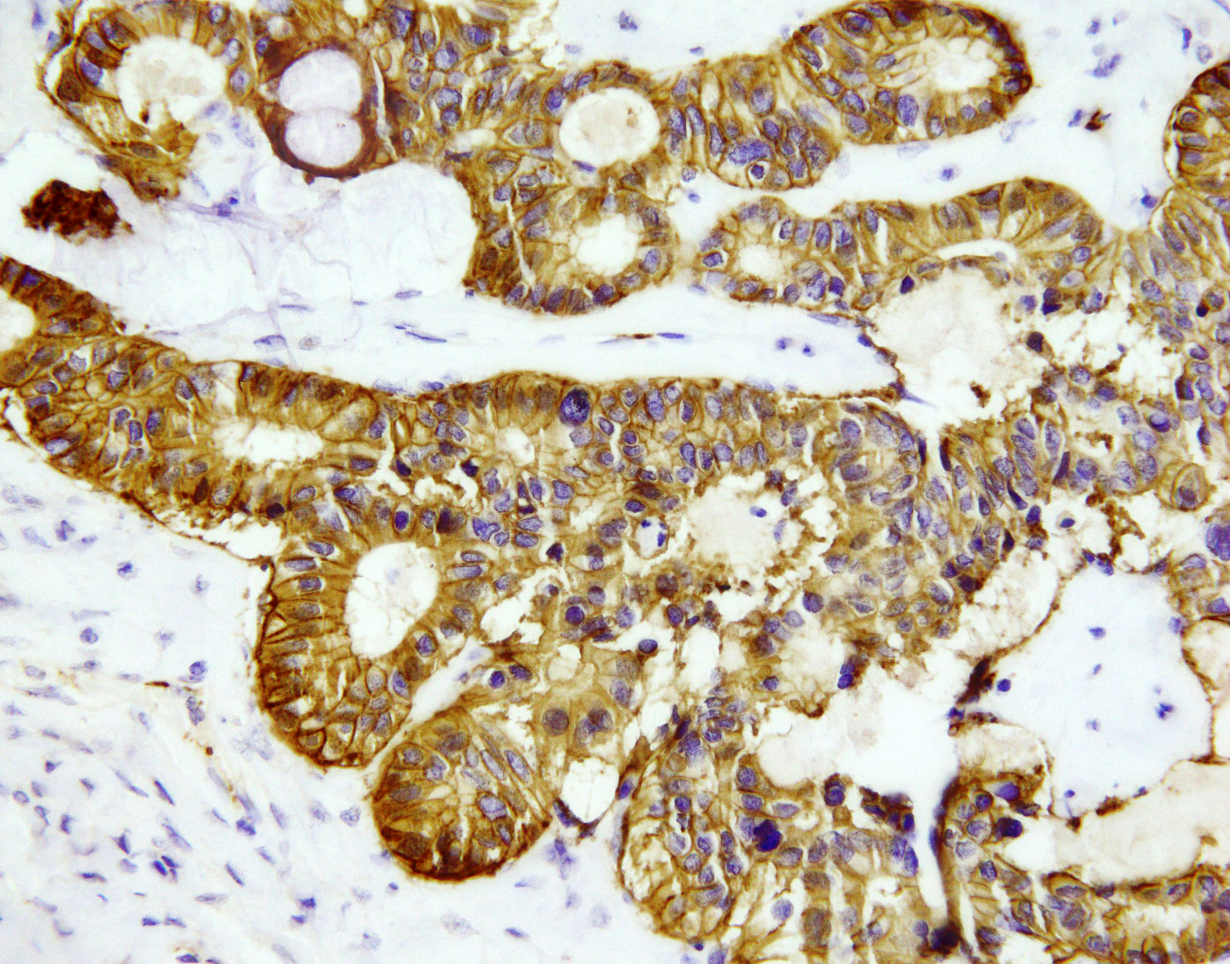
NET focus in teratoma SSTR 2 expression, zoom 40x. Immunohistochemical staining with somatostatin receptor SSTR2: all cells show positive membrane reaction.

**Figure 3:**
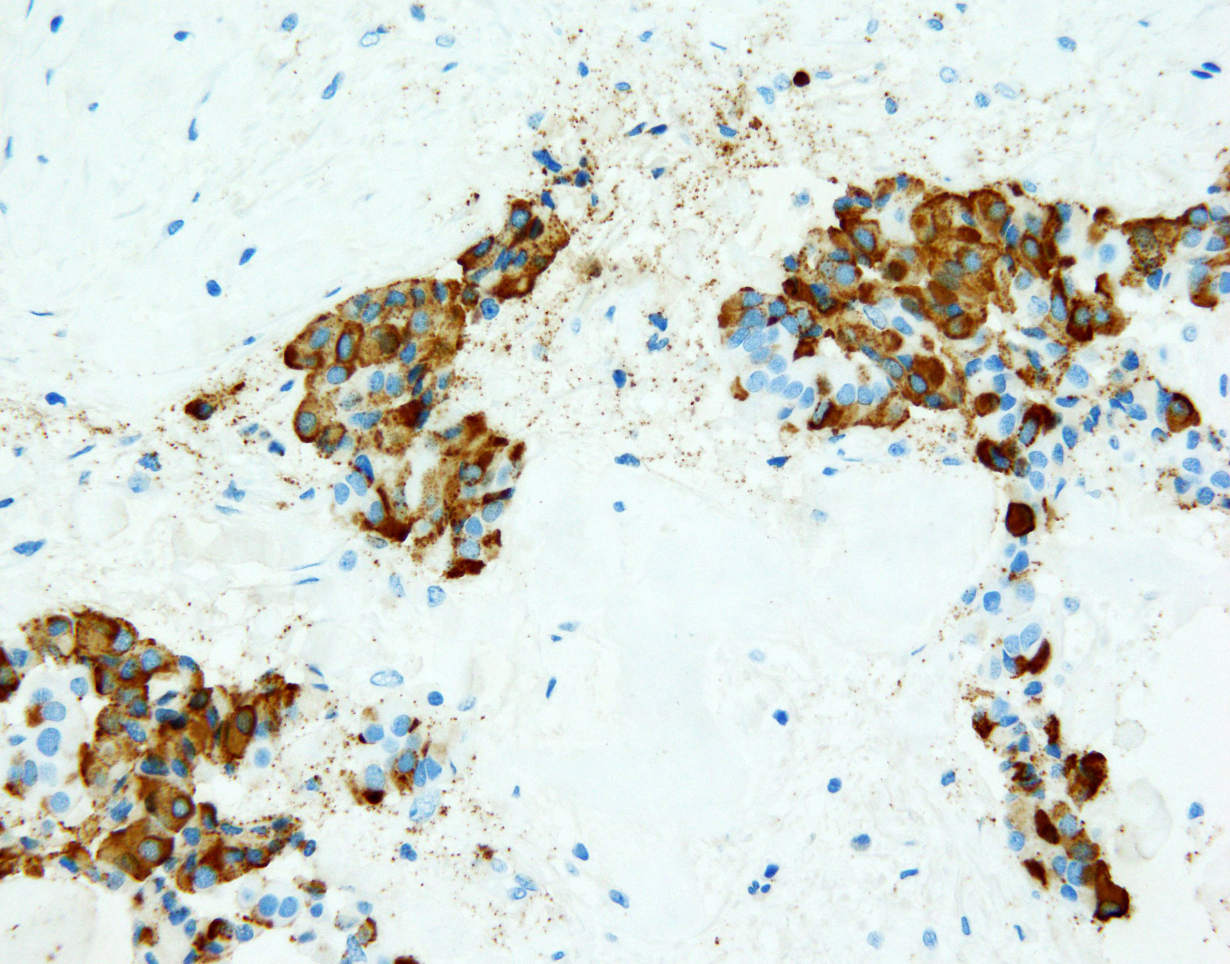
NET focus in teratoma. Immunohistochemical staining (zoom 40x) with chromogranin A: around 85% of cells show positive granular, cytoplasmic reaction.

**Figure 4:**
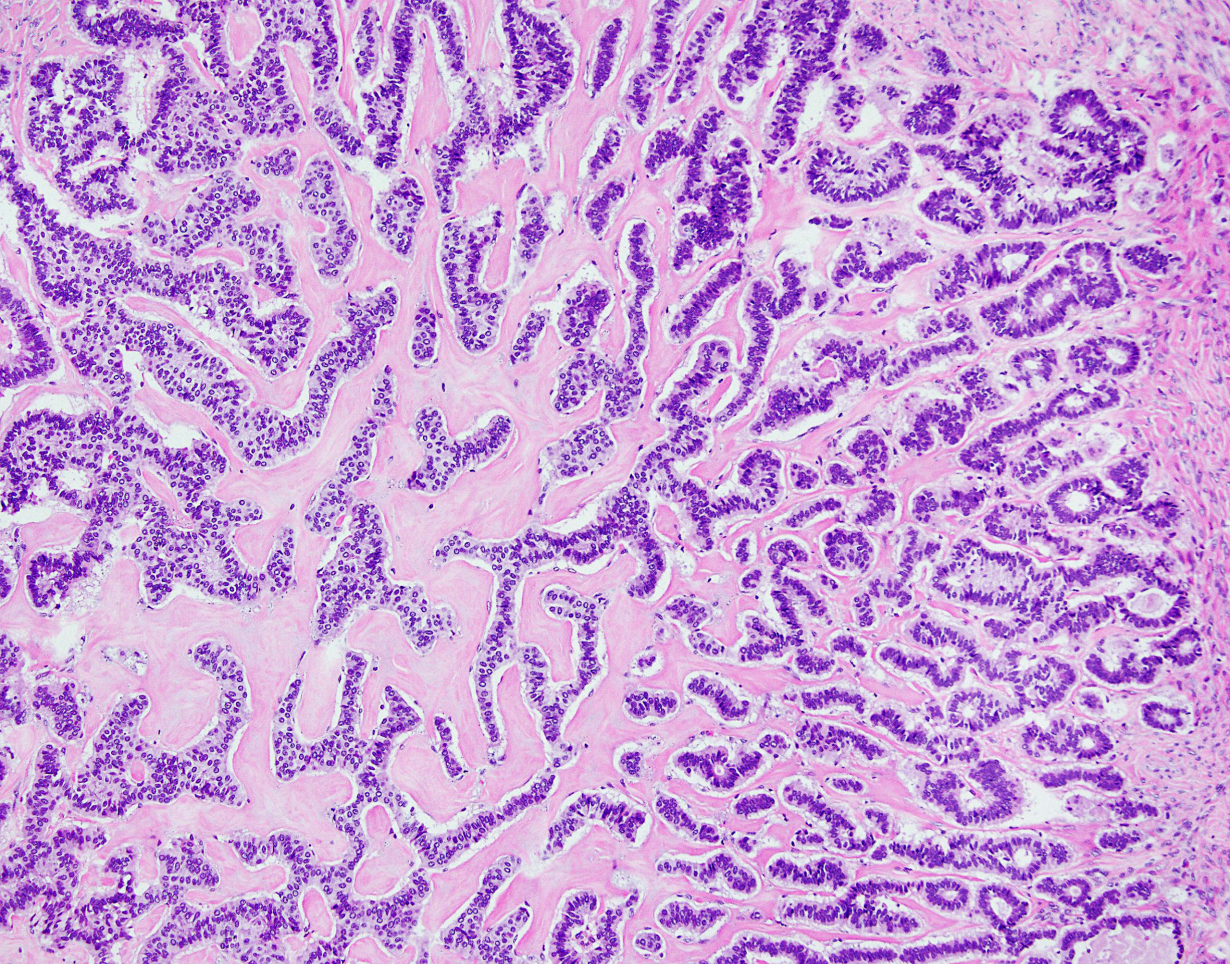
NET foci in teratoma. Hematoxylin-eosin staining, zoom 10x. Long parallel ribbons and small islands and nests of cells with eosinophilic cytoplasm and oblong nuclei oriented perpendicular to main axis of ribbons.

**Figure 5:**
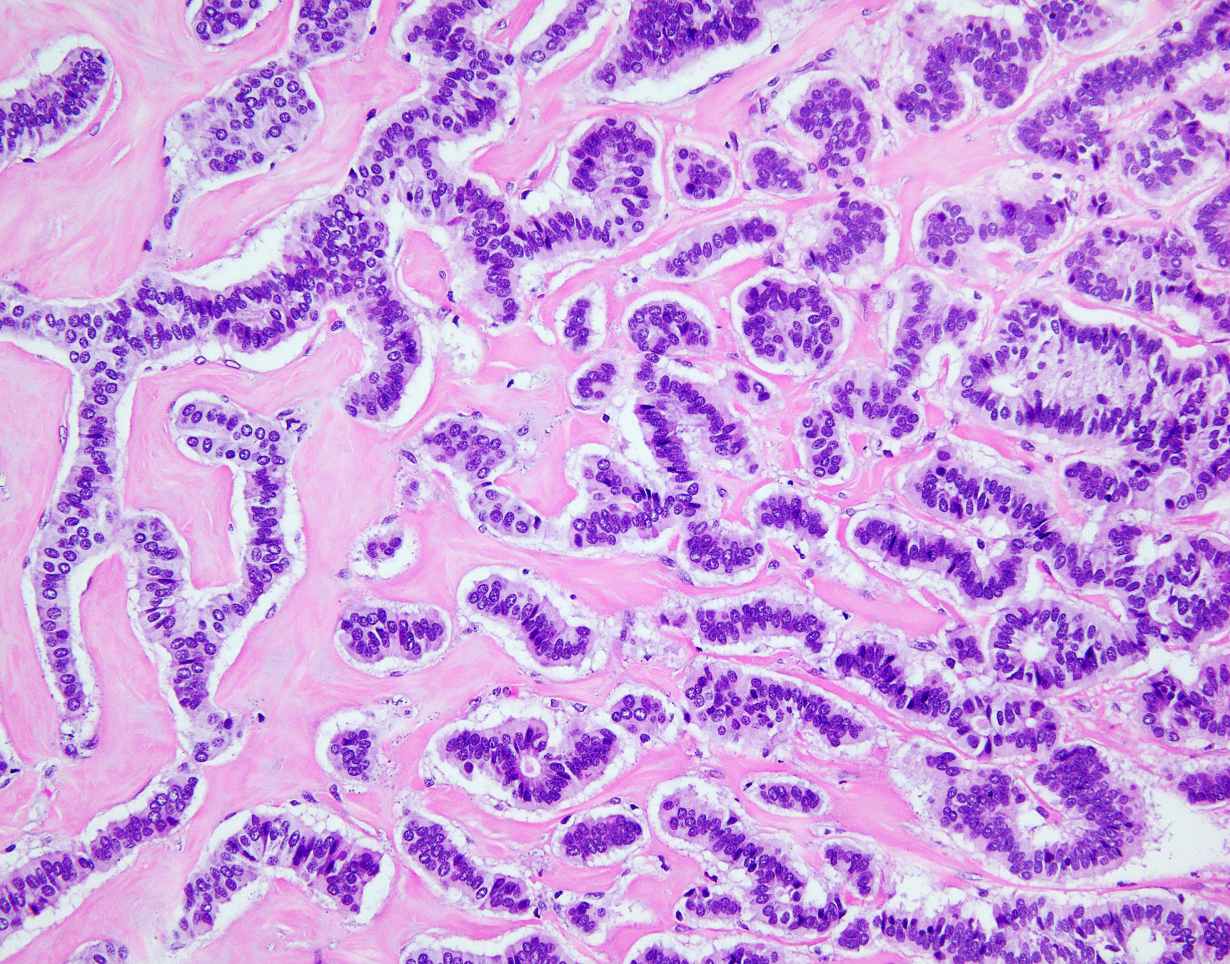
NET foci in teratoma. Hematoxylin-eosin staining, zoom 20x. Long parallel ribbons and small islands and nests of cells with eosinophilic cytoplasm and oblong nuclei oriented perpendicular to main axis of ribbons.

In 8 specimens, Ki-67 was determined and ranged from 2% to 9%. Forty percent (n=4) were classified as NET G1 and another 40% (n=4) as NET G2. In histological examination, synaptophysin and chromogranin were positive in 9 patients (**Figures 1**–**5**) In one patient, no histopathological staining was performed, but the morphology of the neuroendocrine component was typical. Serum chromogranin A (CgA) level was determined postoperatively in 7 patients after a neuroendocrine component was found in the histopathological examination; in 4 of them, CgA levels were elevated even several months after the surgery. Total time of observation ranges from 4 to 59 months, with a mean value of 29.7 ± 21.28 months. All patients who remain in observation are progression-free. Two patients at premenopausal age were lost to follow up after more than 3 years of observation.

All results are summarized in **Table 1**.

**Table 1:** Characteristics of patients and excised teratomas.

	Patients at premenopausal age (n = 7)	Patients at postmenopausal age (n = 3)	Total (n=10)
**Age at diagnosis mean +/- SD, years (range)**	**33.14±9.06 (19-47)**	**65.33±10.4(57-77)**	**42.8±17.91 (19-77)**
**Months in observation mean +/- SD (range)**	**29.00±22.00 (4-55)**	**31.33±24.09 (15-59)**	**29.70±21.28 (4-59)**
**Histopatological types of NETs teratoma**	NET within mature ovarian teratoma (n=4) Monodermal teratoma with strumal carcinoid (n=3)	**NET within mature ovarian teratoma (n=3)**	NET within mature ovarian teratoma (n=7) Monodermal teratoma with strumal carcinoid (n=7)
**Side of ovary tumor**	Left (n=5) Right (n=2)	Left (n=1) Right (n=2)	Left (n=6) Right (n=4)
**Size of NEN lesion mean +/-SD [mm]**	**4.46±2.79 (n=6)**	**2.20 (n=1)**	**3.50±2.88 (range 2-9)**
**Chromogranin A level mean +/-SD [ng/ml]**	**69.25±27.58 (n=4)**	**195 (n=1)**	**94.4±61.10 (range 30-195)**
**Size of NEN lesion [mm]**	**7±2.83 (n=2)**	**12±4.24 (n=2)**	**4.46±2.79 (range 5-9)**
**Chromogranin A level [ng/ml]**	**5.34±2.71 (n=5)**	**10.0±10.04 (n=2)**	**6.67±5.18 (n=7)**

n, numbers of cases; NET, neuroendocrine tumor; SD, standard deviation; mm, millimeters.

## Discussion

In recent years, the reported incidence of neuroendocrine tumors has been increasing, probably due to the better sensitivity of diagnostic tests and more frequent staining of chromogranin A and synaptophysin during the histopathological examination. Out of 10 cases, 50% (n=5) were detected over the last 2 years, while the remaining 50% (n=5) in the previous 5 years. The number of NETs primary to the gynecologic tract remains limited, with insufficient retrospective and prospective data (12), however the first case of a carcinoid ovarian teratoma was reported in 1939 (13).

Mature cystic teratomas typically occur in young women, while NET within ovarian teratoma is usually found in the postmenopausal age (14). However, in our study, only 30% (n=3) of patients were diagnosed after the menopause. Moreover, all patients diagnosed with ovarian carcinoid were in the premenopausal age (**Table 1**).

Carcinoid tumors of the ovary may be primary or metastatic. The morphological features of cells of metastatic carcinoids are similar to those which primarily arise in the ovary. Features that indicate the primary nature of the ovarian carcinoid are simultaneous presence of teratomatous elements in that tumor and unilaterality. Metastatic carcinoids are often bilateral, may be multinodular in each ovary, teratomatous elements are not present and extensive vascular invasion may be present.

Primary ovarian carcinoids are classified into four categories: insular, trabecular, strumal and mucinous, with insular being the most common one (15). Out of them, insular type most commonly results in carcinoid syndrome and is the most commonly observed in Western countries, whereas trabecular and strumal NETs are primarily reported in Japan (16). All types are frequently associated with a mature cystic teratoma or mucinous tumor. The current study includes both insular and strumal lesions; neither trabecular nor mucinous NETs of the ovary were found among our patients.

Primary ovarian NET may occur on top of ovarian teratoma or in an otherwise normal ovary. The majority of ovarian teratomas, same as ovarian carcinoids within teratomas and their coexisting variants are found in routine abdominal ultrasound, which is in line with findings of our study but may be surprising considering the large size of those tumors.

If symptoms are present, they vary from non-specific complaints such as constipation or abdominal pain to emergency situations arising from tumor mass, as was seen in one of our patients. The biggest available study comes from Japan, where Soga et al. analyzed patients from 273 articles; 329 cases in total. Out of them, approximately half (43%) were pure ovarian NETs while 57% were associated with a teratoma. Tumors associated with mature teratomas were smaller, less frequently associated with metastases and had better 5-year survival [ (17). Other authors also report excellent outcome after treating early-stage ovarian NETs arising in mature teratomas confined to one ovary with surgery alone (12, 18). Yamasaki et al. suggested that NETs arising in mature teratomas have low malignancy potential (19) and the aggressive course or metastasis was uncommon. In 2011, the Society of Gynecologic Oncology (SGO) issued a document on management of neuroendocrine tumors of the gynecologic tract. The surgery if radical can be considered as a curative without necessity of any adjuvant therapy (12). However, the prognosis and clinical behavior of NETs associated with ovarian teratomas have not been assessed yet, mainly due to its rarity.

According to the literature, approximately one third of all patients with neuroendocrine tumors within ovarian teratoma would present typical symptoms of carcinoid syndrome such as flushing and diarrhea due to bypassing the portal circulation by ovarian venous drainage (20). Some cases of hormonally active ovarian NETs were reported in Japan, however, they were not associated with ovarian teratoma (16, 21–24). Very few cases of primary ovarian NET associated with teratoma were complicated by severe course of the carcinoid syndrome (25, 26). There are also single reports on ACTH-secreting carcinoid components located in an ovarian mature teratoma, manifesting with hypercortisolemia without circadian rhythm with a lack of cortisol suppression in dexamethasone tests (27). In presented study, none of the lesions had hormonal activity and thus, the patients did not experience symptoms related to the overproduction of hormones. Another rare but important issue is possibility of ovarian teratomas recurrence (28, 29). We had not observed relapse of tumors in our cohort.

Immunohistochemical staining of ovarian carcinoids include evaluation of neuroendocrine markers such as synaptophysin, chromogranin A and the proliferative activity of Ki-67/MIB1. In the case of clinical presentation of hormonal syndromes, the expression of hormones, particularly insulin, gastrin and serotonin, ACTH should be assessed. In case of all ovarian tumors, their metastatic nature should be ruled out. In few studies the utility of markers indicating the origin of neuroendocrine tumors, such as cytokeratin CK20, CK7, thyroid transcription factor (TTF-1) and CDX-2 in ovarian neuroendocrine tumors were determined. Rabban et al. examined the site of origin in 26 NETs (16 primary and 10 metastatic from midgut). Teratomatous elements were present in association with 10/16 primary ovarian NETs, whereas none were present in metastatic ones. Clinicopathologic features such as unilaterality, absence of multinodular growth, early stage, presence of teratomatous elements, and small size were the most helpful in suggesting a primary origin for an ovarian carcinoid tumor (10). Study of Gungor et al. states that metastatic carcinoid inside the ovary is always bilateral (30). However, to our knowledge, no case of primary bilateral NETs with ovarian teratoma has not been reported yet, as was not seen in our population. However, differential diagnosis should include metastatic neuroendocrine tumors (metastases from gastrointestinal tract are usually CK20+ and CDX2+ and metastases from lungs are usually CK7+ and TTF1+), granulosa cell tumors (which is inhibin+ and calretinin+), poorly differentiated primary or metastatic adenocarcinomas (synaptophysin and chromogranin negative), Brenner tumors and androblastoma (9).

For follow-up the abdominal and pelvic imaging should be performed as well as chromogranin A (CgA) concentration assessed. It is important to remember that false-positive elevation of CgA is presented in many clinical settings like impaired renal function, chronic inflammations, chronic atrophic gastritis (type A), use of Proton Pump Inhibitors, glucocorticosteroids or others (11). For that reason, an isolated increase in CgA values require excluding other than NET causes of its elevation as observed in some of our patients in whom CgA increase was not accompanied by the disease relapse.

According to literature several other biochemical markers like circulating tumor cells, multiple transcript analysis, microRNA profile were tested as the tools for more accurate NET follow-up (31). Among them NETest (PCR based analysis of 51 different NET-related transcripts) seems to be the most promising in gastro-entero-pancreatic (GEP), and pulmonary NETs predicting radiologicalrelapse with 94% accuracy and 100% sensitivity (32). In the case of reproductive system NETs, the efficacy of NETest, has not been evaluated, yet.

In case of potentially inoperative or disseminated disease, there is a possibility, like in GEP-NETs, of SSTR assessment in the tumor tissue. It may be crucial for a further therapy planning (7, 11). The somatostatin analogues are recommended in treatment of well or moderately differentiated G1 and G2 GEP-NETs with good SSTR expression, while PRRT may be used after progression on somatostatin analogues in G1 and G2 GEP-NETs and additionally in G3 tumors either with good SSTR expression.

## Data Availability Statement

The original contributions presented in the study are included in the article/supplementary material. Further inquiries can be directed to the corresponding author.

## Ethics Statement

The study protocol was approved by the Local Ethics Committee of the Jagiellonian University in Krakow. Written informed consent for participation was not required for this study in accordance with the national legislation and the institutional requirements.

## Author Contributions

MO, Data collection, data analysis, manuscript drafting, manuscript editing and approval, study coordination. AS-S, Data collection, data analysis, manuscript drafting, manuscript editing and approval. HO and MU-B, Data collection, manuscript drafting, manuscript editing and approval. AG-J, Data analysis, manuscript editing and approval. AH-D, Data analysis, manuscript drafting, manuscript editing and approval. All authors contributed to the article and approved the submitted version.

## Conflict of Interest

The authors declare that the research was conducted in the absence of any commercial or financial relationships that could be construed as a potential conflict of interest

## Publisher’s Note

All claims expressed in this article are solely those of the authors and do not necessarily represent those of their affiliated organizations, or those of the publisher, the editors and the reviewers. Any product that may be evaluated in this article, or claim that may be made by its manufacturer, is not guaranteed or endorsed by the publisher.
